# Toward real‐world deployment of machine learning for health care: External validation, continual monitoring, and randomized clinical trials

**DOI:** 10.1002/hcs2.114

**Published:** 2024-10-14

**Authors:** Han Yuan

**Affiliations:** ^1^ Centre for Quantitative Medicine Duke‐NUS Medical School Singapore Singapore

**Keywords:** machine learning, real‐world deployment, external validation, continual monitoring, randomized clinical trials

## Abstract

In this commentary, we elucidate three indispensable evaluation steps toward the real‐world deployment of machine learning within the healthcare sector and demonstrate referable examples for diagnostic, therapeutic, and prognostic tasks. We encourage researchers to move beyond retrospective and within‐sample validation, and step into the practical implementation at the bedside rather than leaving developed machine learning models in the dust of archived literature.

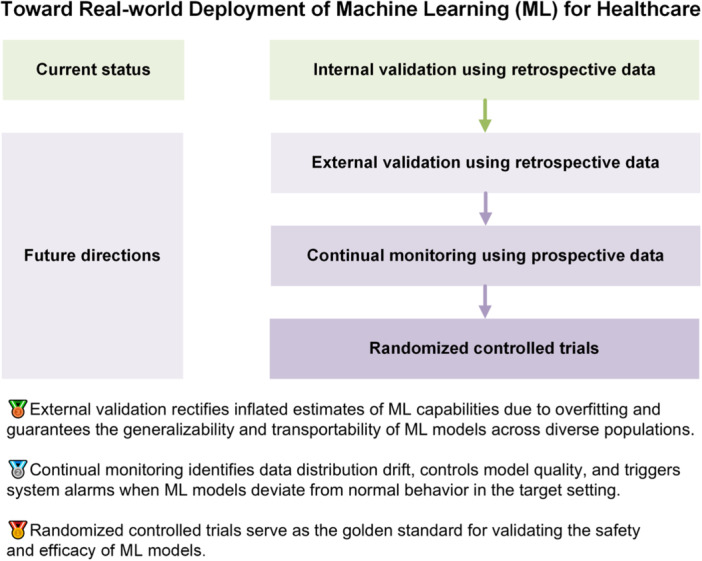

**Abbreviations**
FDA: US Food and Drug Administration
ML: machine learning
RCTs: randomized controlled trials

## 1 OVERVIEW

Machine learning (ML) has been increasingly used for tackling various diagnostic, therapeutic, and prognostic tasks owing to its capability to learn and reason without explicit programming [1]. Most developed ML models have had their accuracy proven through internal validation using retrospective data. However, external validation using retrospective data, continual monitoring using prospective data, and randomized controlled trials (RCTs) using prospective data are important for the translation of ML models into real‐world clinical practice [2]. Furthermore, ethics and fairness across subpopulations should be considered throughout these evaluations.

## 2 EXTERNAL VALIDATION

Different from internal validation, which evaluates the performance of ML using a subset of the original datasets, external validation assesses ML models in contexts that may vary subtly or considerably from the one in which they were developed [3]. External validation serves to rectify inflated estimates of ML capabilities owing to overfitting and guarantees the generalizability and transportability of ML models across diverse populations [4]. For external validation, researchers can leverage the abundant resources of publicly accessible databases such as PhysioNet [5]. Three external validation scenarios are recommended after identifying a suitable database with a sufficient sample size to guarantee testing robustness [6]. The first involves directly deploying the trained ML models on external data to simulate a brand‐new scenario without previous data [6]. The second entails using a large training data set from the new scenario to fine‐tune the developed models, simulating that ample data have been collected in the external context [7]. The third scenario represents an intermediate situation wherein new data are gradually fed into the ML models to simulate a scenario where the models are deployed in a new setting, new data are incrementally collected, and the models are updated iteratively with the newly collected data [8]. Most existing studies have focused on the direct deployment of ML models for diagnostic, therapeutic, and prognostic tasks [9]. Holsbeke et al. [10] deployed previously published diagnostic ML models for detecting adnexal mass malignancy across multiple medical centers in different countries with different population characteristics. For external validation of therapeutic ML models, a pertinent reference is a study investigating the survival benefits of adjuvant therapy in breast cancer where researchers evaluated ML models, which were originally developed using populations from the United Kingdom, in clinical settings in the United States [11]. In the realm of prognostic tasks, Clift et al. [12] offered a comprehensive approach to externally validate ML models in the context of predicting the 10‐year risk of breast cancer‐related mortality, detailing methods for sample size calculation, population identification, outcome definition, and performance evaluation. In addition to assessing model performance, similarity between the original training datasets and external validation datasets can be quantified to enable the elucidation of performance degradation and further identify potential avenues for model enhancement [13].

## 3 CONTINUAL MONITORING

Following large‐scale external validation using retrospective data, the subsequent step in implementation is prospective evaluation in the specific setting where an ML model is to be deployed [14]. Specifically, ML models receive prospective data, make predictions accordingly, and are evaluated within a predefined time frame [14]. Compared with the first step, continual monitoring is used to identify data distribution drift, control model quality, and trigger system alarms when an ML model deviates from its normal behavior in the target setting [15]. Because the operation and monitoring of ML models are mainly conducted by clinical professionals, developers should focus on translation of the developed ML models into a user‐friendly clinical practice. The first aspect is the operation of ML models in an offline hospital system where allocated computation resources would be limited for low latency in responding to other functions inside the system. The second aspect is the development of a secure and privacy‐aware maintenance method for quickly addressing potential technical collapses while minimizing direct access to patients' private data. The last aspect is the development of a user‐friendly interface such as an Android app [16] or web‐based software [17] that facilitates the use of ML models by health care professionals and comprehends their suggestions. It should be emphasized that the application of ML in a prospective clinical setting should be designed to operate independently from, and not interfere with, existing clinical decision‐making processes. This precaution is necessary to avoid any potential adverse impact on the existing health care quality. Exemplary continual monitoring of therapeutic ML models can be seen in the work of Wissel et al. [18]. Those authors conducted a prospective, real‐time assessment of ML‐based classifiers for epilepsy surgery candidacy at Cincinnati Children's Hospital Medical Center. To mitigate any risks associated with ML classifiers, patients who were deemed appropriate surgical candidates by the algorithm were subjected to manual review by two expert epileptologists, with final decisions on their surgical candidacy confirmed via a comprehensive expert chart review. A critical insight from the study was that effective monitoring necessitates a synergistic collaboration between clinicians, who provide essential medical expertize, and information technology professionals, who contribute research and operational knowledge [19, 20]. Assuming that an ML tool demonstrates accurate prospective diagnostic capabilities in the target setting, its developers should pursue approval for further RCTs from administrative ethics committees.

## 4 RANDOMIZED CONTROLLED TRIALS

The last step toward the real‐world implementation of ML tools is classic four‐phase RCTs. To ensure safety in real‐life scenarios, absolutely ML‐based interventions are likely to be avoided. We recommend designing RCTs to compare the accuracy and diagnosis time for clinicians with ML models (intervention group) and without ML models (control group) [21, 22, 23]. For instance, He et al. [24] implemented RCTs to demonstrate that ML‐guided workflows reduced the time required for sonographers and cardiologists in the diagnoses of left ventricular ejection fraction. Specifically, the first step in RCTs is to seek ethical approval from an institutional review board to ensure that the RCTs comply with ethical standards and regulations. Then, researchers can proceed with Phase I of the clinical trial to assess safety (whether the introduction of an ML model distracts clinicians and impairs their diagnoses) and to identify specific scenarios in which ML should be used. In Phase II, a few hundred patients are recruited to assess whether statistically significant improvements result from the use of ML tools in clinicians' diagnoses. In Phase III, several hundred or even several thousand patients are recruited to validate the safety and effectiveness of the ML tool, demonstrating its superiority over other existing solutions. If the ML tool receives approval from the administrative agency after Phase III, researchers can then investigate its effectiveness and safety in a wider range of patients in Phase IV. Upon demonstrating efficacy through rigorously conducted RCTs, ML tools can receive approval from national regulatory agencies such as the US Food and Drug Administration (FDA) for commercialization [25]. A paradigmatic illustration of RCTs for diagnostic ML models can be found in the research by Titano et al. [26]. Those authors developed three‐dimensional convolutional neural networks to diagnose acute neurological events using head computed tomography images. The efficacy and efficiency of ML models were subsequently validated in a randomized, double‐blind, prospective trial. For therapeutic ML models, we suggest referring to Nimri et al. [27]. The researchers conducted multicenter and multinational RCTs to compare ML with physicians from specialized academic diabetes centers in optimizing insulin pump doses. In the realm of prognostic ML models, researchers from the Mayo Clinic implemented RCTs to assess the effectiveness and efficiency of ML models in predicting 1‐year occurrence of asthma exacerbation [28]. A detailed guideline for conducting RCTs on ML for health care could benefit from the FDA's Policy for Device Software Functions and Mobile Medical Applications [29], which includes specific provisions for medical applications that apply ML algorithms [30].

## 5 TOWARD REAL‐WORLD DEPLOYMENT

Alongside population‐level evaluations, there has been burgeoning awareness about the ethical implications of ML models, which have been revealed to diagnose, treat, and bill patients inconsistently across subpopulations [31]. Therefore, it is imperative to ensure equity of patient outcomes, model performance, and resource allocation across subpopulations in the real‐world deployment of ML models [31, 32, 33]. Thompson et al. [34] proposed a reference framework to mitigate ML biases using two recalibration modules. The first module adjusted the decision cutoff threshold for subpopulations affected by bias, and the second recalibrated model outputs, enhancing their congruence with the observed events. Chen et al. [31] systematically summarized the path toward deployment of ethical and fair ML in medicine, which includes a diverse subpopulation collection using federated learning, fairness principles, operationalization across health care ecosystems, and independent regularization and governance of data and models to avoid disparities. Apart from various performance assessments, clinicians' endorsement and patients' approval of ML models should be thoroughly integrated into the evaluation processes [31, 35].

In this commentary, we elucidate three indispensable evaluation steps toward the real‐world deployment of ML within the health care sector and provide examples of diagnostic, therapeutic, and prognostic tasks. In light of these, we encourage researchers to move beyond retrospective and within‐sample validation and toward the practical implementation at the bedside rather than leaving developed ML models buried within the archived literature.

## AUTHOR CONTRIBUTIONS

**Han Yuan**: Conceptualization (lead); data curation (lead); formal analysis (lead); investigation (lead); methodology (lead); writing—original draft (lead); writing—review and editing (lead).

## CONFLICT OF INTEREST STATEMENT

The author declares no conflict of interest.

## ETHICS STATEMENT

This study is exempt from review by the ethics committee because it did not involve human participants, animal subjects, or sensitive data collection.

## INFORMED CONSENT

Not applicable.

## Data Availability

Data sharing not applicable to this article as no datasets were generated or analyzed during the current study.
